# Multimodal Ultrasound in Evaluating Radiotherapy Efficacy for Vaginal Squamous Cell Carcinoma: A Case Report

**DOI:** 10.2174/0115734056465978260626093538

**Published:** 2026-07-07

**Authors:** Chanjuan Peng, Ru Chen, Jiahui Yu, Hongyun Zhang, Xiaoting Chen, Jiang Zhu

**Affiliations:** 1 Department of Ultrasound, Women's Hospital School of Medicine, Zhejiang University, Hangzhou, China;; 2 Zhejiang Provincial Key Laboratory of Precision Diagnosis and Therapy for Major Gynecological Diseases, Women's Hospital, Zhejiang University School of Medicine, Hangzhou, China;; 3 Zhejiang Cancer Hospital, Hangzhou Institute of Medicine (HIM), Chinese Academy of Sciences, Hangzhou, Zhejiang 310022, China;; 4 Department of Ultrasound, Yueqing People's Hospital, Wenzhou, China

**Keywords:** Vaginal neoplasms, Ultrasonography, Contrast-enhanced ultrasound, Multimodal ultrasound, MRI, Radiotherapy efficacy

## Abstract

**Background:**

Vaginal malignancy is rare and carries a poor prognosis. MRI is the reference standard for staging and follow-up, but cost and availability are limiting. We describe the use of high-frequency transrectal ultrasound (HF-TRUS) for pre-treatment mapping and early response assessment of primary vaginal cancer.

**Case Presentation:**

A 65-year-old woman presented with post-menopausal bleeding for 20 days. Histology of the right vaginal fornix revealed high-grade squamous intraepithelial lesion (HSIL). Clinical stage cT1N0M0 poorly differentiated carcinoma was confirmed, and curative radiotherapy was planned. HF-TRUS (12 MHz linear array, sagittal and axial planes) showed two heterogeneous hypoechoic nodules on the anterior vaginal wall. Gel filling in the vaginal canal created an effective acoustic window, which significantly improved the delineation of the vaginal wall layers and the lesion boundaries. After 45 Gy external-beam radiotherapy, the nodules decreased by 15.2% and 18.2%, respectively; at 6 months post-completion of brachytherapy, one nodule had virtually disappeared. Doppler signal decreased concurrently. MRI at the same time-points provided identical size regression data.

**Conclusion:**

This case validates HF-TRUS with contrast enhancement as a reliable MRI alternative for monitoring radiotherapy in vaginal cancer. The ultrasound accurately tracked tumor size and blood flow changes, with results matching MRI. This quick, clinic-based technique fills a critical gap when MRI is not available or contraindicated.

## 1 INTRODUCTION

Primary malignant vaginal tumors are exceedingly rare, constituting only 0.3% of malignancies in the female genital tract, with an annual incidence of approximately 1 in 100,000 women [1]. In the United States, around 2,160 new cases are diagnosed each year, leading to an estimated 770 deaths. Histologically, squamous cell carcinoma is the predominant type (83.4%), followed by adenocarcinoma (9.3%), malignant melanoma (2.6%), and vaginal sarcoma (2.6%). Other rare pathologic variants have also been documented, including small cell carcinoma, lymphoma, carcinoid tumor, and undifferentiated carcinoma [2]. Given the rarity of primary vaginal cancer, metastases from such sites as the endometrium, cervix, vulva, ovary, breast, colorectum, and kidney are often included in the scope of observation and differential diagnosis [3]. It therefore imposes requirements of investigation to exclude metastatic disease, where imaging assumes a decisive role.

MRI is currently considered the gold standard for local staging and treatment monitoring in vaginal cancers due to its superior soft tissue resolution [4]. However, MRI may be contraindicated in some patients (*e.g*., those with claustrophobia, metallic implants, or renal impairment) and is not always readily accessible for frequent follow-up assessments. High-frequency transrectal ultrasound (HF-TRUS) with contrast-enhanced ultrasound (CEUS) has emerged as a potential alternative, offering real-time, high-resolution imaging of vaginal lesions [5]. While ultrasound is well-established in other gynecologic applications, its role in monitoring radiotherapy response for primary vaginal cancer remains poorly characterized, with limited data comparing its performance to MRI.

Here, we present a case of primary vaginal squamous cell carcinoma monitored with HF-TRUS and CEUS during radiotherapy, with treatment response consistent with MRI. This case demonstrates the utility of ultrasound-based imaging as an alternative when MRI is unavailable or contraindicated, addressing a gap in the literature on non-MRI approaches for treatment monitoring in vaginal cancer.

## 2 CASE PRESENTATION

### 2.1 General Information

A 65-year-old presented with 20 days of post-menopausal vaginal bleeding occurring 17 years after her last natural menstruation. The episode began without trauma or hormone use: the first 48 h required sanitary pads, after which only spotting was noted. She denied prior post-menopausal bleeding, discharge, dyspareunia, or weight loss and had not attended gynaecological screening for five years. Surgical history included laparotomic myomectomy and left oophorectomy 20 years earlier; medical history was otherwise unremarkable. Her mother died of cervical cancer at 58 years.

The patient was a non-smoker with a BMI of 24.67 kg/m^2^. She was married with two children (G2P2) and reported no recent high-risk sexual behavior. There was no history of diethylstilbestrol (DES) exposure.

On examination, the external genitalia were normal; a brownish serosanguinous discharge was present. A 4 × 3 cm irregular, friable, firm mass arose from the right posterolateral wall of the middle-to-lower third of the vagina, 2 cm distal to the fornix and 1.5 cm proximal to the introitus. No parametrial or rectal involvement was palpable.

**Fig. (1):**
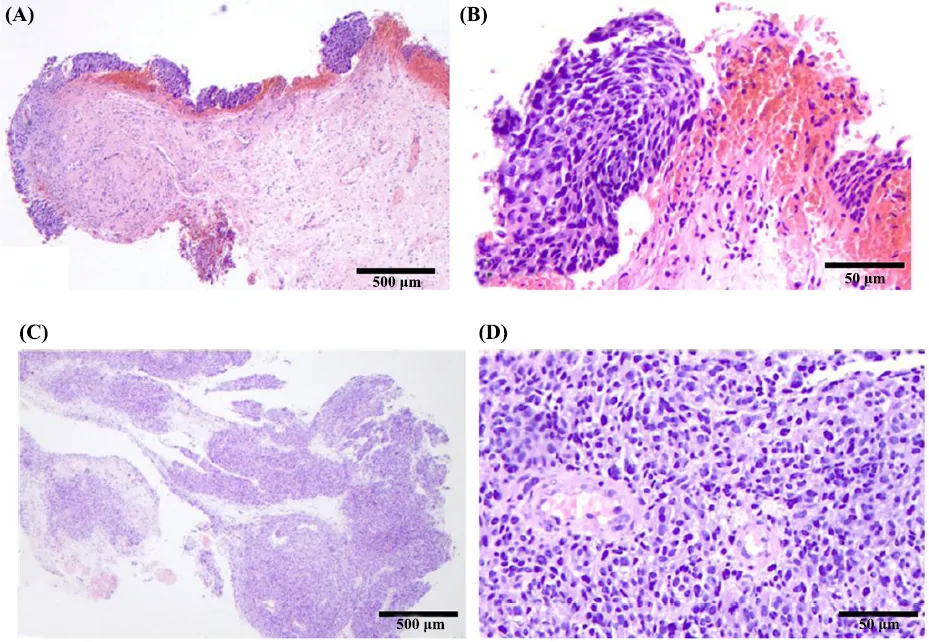
Vaginal squamous cell carcinoma. (**A**) and (**B**) Photomicrographs of H&E-stained sections at 40× and 200× magnification, respectively, showing squamous epithelial cells with severe atypical hyperplasia, progressively transforming into in situ squamous cell carcinoma (A Scale bar: 500μm; B Scale bar: 50μm). (**C**) and (**D**) Photomicrographs of H&E-stained sections at 40× and 200× magnification, respectively, illustrating the invasive growth pattern of the tumor, infiltrating the stroma. The tumor cells exhibit irregular shapes, enlarged and irregular nuclei, with prominent hyperchromasia (C Scale bar: 500μm; D Scale bar: 50μm).

Laboratory tests: CA125, CA19-9, CA15-3, CEA, AFP, and NSE were normal; SCC antigen was 0.89 ng mL^−1^ (upper limit 1.5), and CA72-4 was 9.73 U mL^−1^ (reference <6.9). Liquid-based cytology showed ASC-US; high-risk HPV E6/E7 mRNA was negative. Punch biopsies under local anaesthesia revealed:(1) Right vaginal fornix – high-grade squamous intraepithelial lesion (HSIL/VAIN III). (2) Right anterior vaginal wall mass – poorly differentiated carcinoma (Fig. **1**).

### 2.2 Pretreatment Imaging

#### 2.2.1 Color Transvaginal Ultrasound

Transvaginal ultrasound revealed an anteverted, atrophic uterus. The endometrial lining was thin (0.4 cm, double-layered) and heterogeneous in echogenicity. A heterogeneous, hypoechoic lesion with irregular borders was identified in the vaginal wall. Color Doppler Flow Imaging (CDFI) demonstrated internal blood flow within the lesion. The adnexa were unremarkable. These findings were consistent with an atrophic uterus and a suspicious vascular lesion of the vaginal wall, raising concern for a primary vaginal tumor.

#### 2.2.2 HF-TRUS

**Fig. (2):**
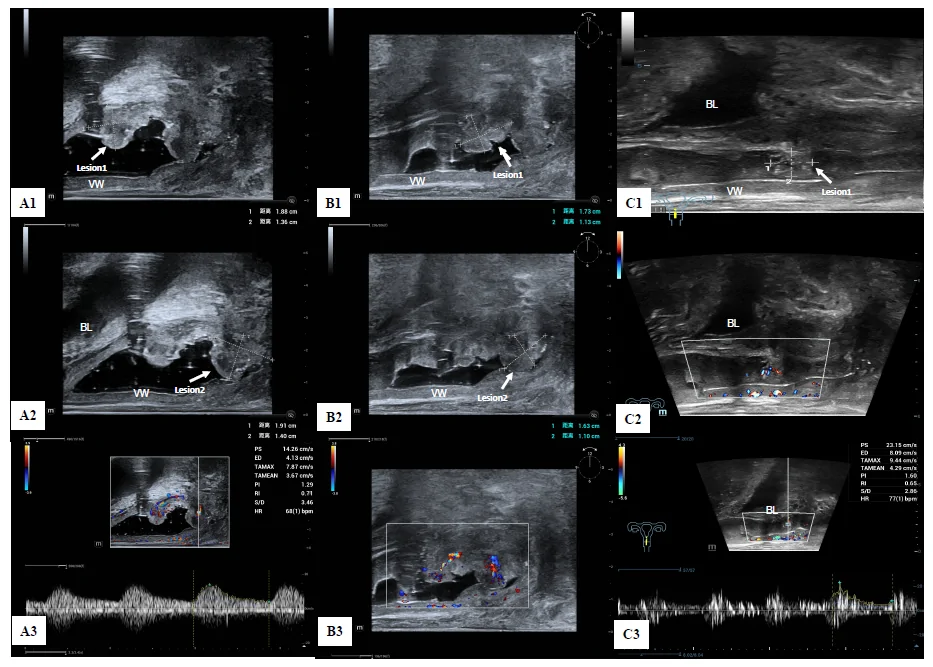
Transrectal high-frequency ultrasound images. (**A1**): Two-dimensional ultrasound image and size measurement of vaginal Lesion 1 before treatment.(**A2**): Two-dimensional ultrasound image and size measurement of vaginal Lesion 2 before treatment.(**A3**): Blood flow image and spectral Doppler measurement of the vaginal Lesion before treatment.(**B1**): Two-dimensional ultrasound image and size measurement of vaginal Lesion 1 after external beam radiotherapy.(**B2**): Two-dimensional ultrasound image and size measurement of vaginal Lesion 2 after external beam radiotherapy.(**B3**): Blood flow image of the vaginal Lesion after external beam radiotherapy. (**C1**): Two-dimensional ultrasound image and size measurement of the vaginal Lesion six months after treatment.(**C2**): Blood flow image of the vaginal Lesion six months after treatment.(**C3**): Spectral Doppler measurement of the vaginal Lesion six months after treatment. BL=bladder, VW=vaginal wall. Flow direction Red: toward transducer, Blue: away from transducer.

HF-TRUS (Fig. **2A**) precisely delineated two heterogeneous, hypoechoic nodules on the anterior vaginal wall, presenting a polypus morphology and measuring approximately 1.9 × 1.4 cm and 1.9 × 1.4 cm, respectively. The nodules displayed irregular and indistinct borders. CDFI detected abundant internal blood flow signals with a resistance index (RI) of 0.74. The muscular layer of the posterior vaginal wall near the lower segment was about 0.97 cm thick, with a poorly defined serosal layer and an unclear boundary with the rectum. These nodules were characterized on imaging by irregular shape, hypervascularity, and infiltrative borders, suggesting a high possibility of malignancy. In addition, thickening of the posterior wall was diagnosed as indicative of tumor involvement.

#### 2.2.3 Contrast-Enhanced Ultrasound (CEUS)

**Fig. (3):**
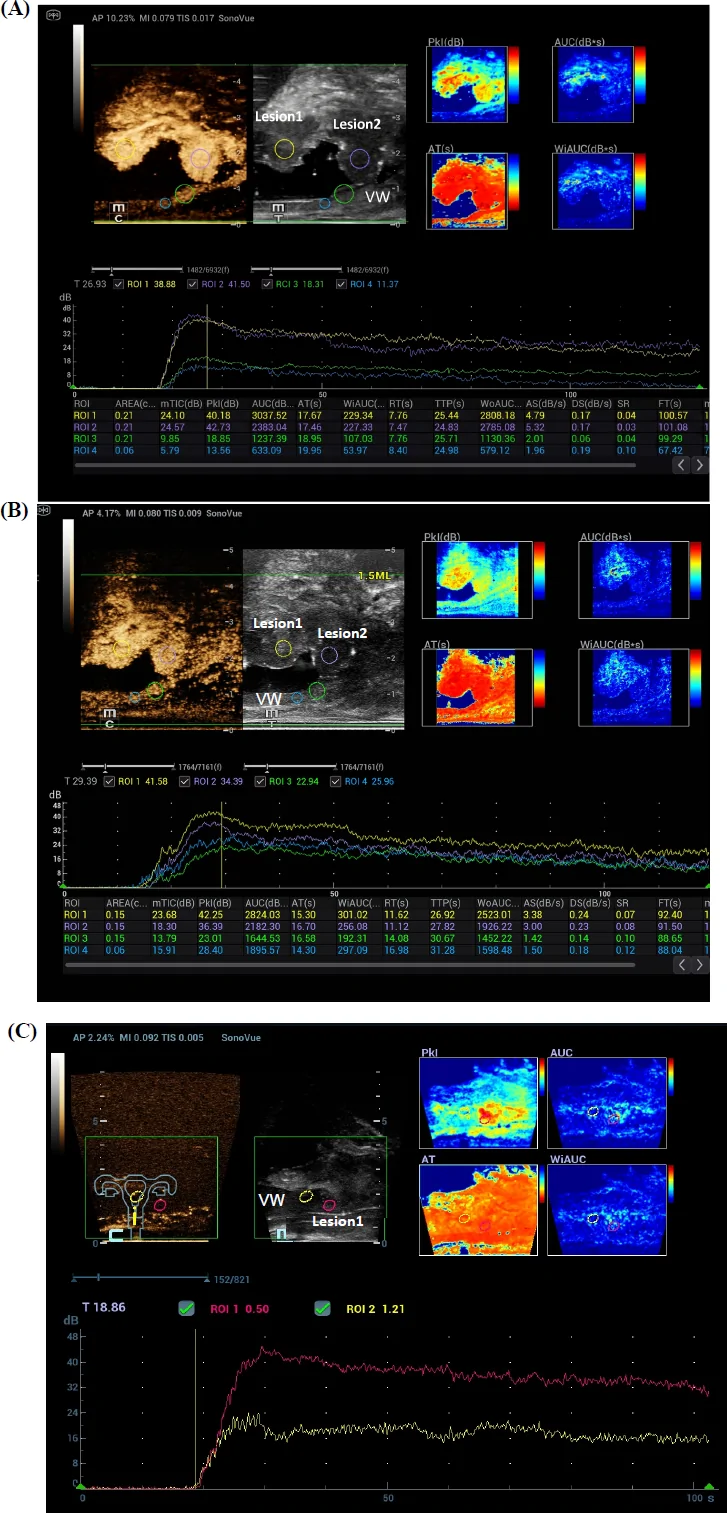
Contrast-enhanced ultrasound for treatment response evaluation of the mass.(**A**): Quantitative analysis of contrast-enhanced ultrasound before treatment. lesion1: yellow ROI 1, Lesion2: purple ROI 2, Thickened VW: Green ROI3, Background: Blue ROI 4. (**B**): Quantitative analysis of contrast-enhanced ultrasound after external beam radiotherapy. lesion1: yellow ROI 1, Lesion2: purple ROI 2, Thickened VW: Green ROI3, Background: Blue ROI 4. (**C**): Quantitative analysis of contrast-enhanced ultrasound for vaginal Lesion, six months after treatment. Lesion1; red ROI 1, Background: yellow ROI 2 VW=vaginal wall,

For contrast-enhanced imaging, 2.4 mL of the ultrasound contrast agent SonoVue^®^ (sulfur hexafluoride microbubbles; Bracco, Milan, Italy) was administered *via* bolus injection at a mechanical index of 0.078. Continuous real-time imaging was performed for 120 seconds following contrast injection. Quantitative analysis of contrast enhancement patterns was conducted using the built-in quantification software of the ultrasound system, generating time-intensity curves and parameters including peak intensity, area under the curve (AUC), and arrival time. Figure **3A** showed both vaginal nodules dramatically hyper-enhanced with noticeable contrast agent filling. The enhancement followed a “fast-in and equal-out” kinetic pattern, and its perfusion morphology resembled a radiating vascular pattern. Quantitative analysis revealed hyper-perfusion in the lesions, which was significantly higher in peak intensity and larger in area under the curve (AUC) than the reference tissue (Table **1**). The CEUS findings indicated highly vascularized lesions, providing convincing imaging evidence that supported the clinical diagnosis.

#### 2.2.4 Pelvic MRI

**Fig. (4):**
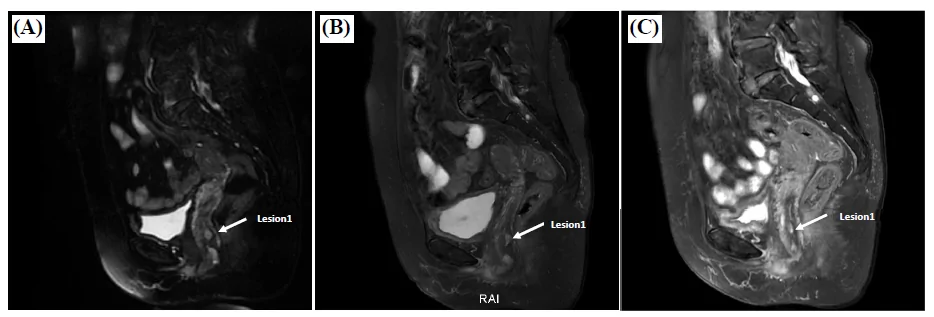
MR Images of the vaginal mass. (**A**): T2-weighted image of the vaginal Lesion 1 before treatment.TR:4140.0, TE:109.3. (**B**): T2-weighted image of the vaginal Lesion 1 after external beam radiotherapy.TR:3687.0, TE:70.0. (**C**): T2-weighted image of the vaginal Lesion 1 six months after treatment. TR:4097.0, TE:67.4.

According to pelvic MRI (Fig. **4A**), there were two nodules in the mid-to-lower vagina (predominantly right-sided). Both showed high DWI signal, low T2WI signal, and mild post-contrast enhancement; the vaginal wall showed marked enhancement, while the uterus showed no abnormal signal or enhancement, suggesting atrophy. The cervix and adnexa were unremarkable, with no enlarged lymph nodes or pelvic fluid. Based on MRI findings of restricted diffusion and enhancement in the vaginal nodules, there was a high possibility of malignancy, with associated inflammatory changes suggested by the concomitant enhancement of the vaginal wall.

### 2.3 Treatment Plan

Diagnosis of the multidisciplinary team (MDT) suggested that the patient had a clinical stage I vaginal malignant tumor (poorly differentiated carcinoma, cT1N0M0), and radiotherapy was the primary treatment for this case. Although radical surgery, such as hysterectomy, pelvic lymphadenectomy, complete vaginectomy, partial vulvectomy, and superficial inguinal lymph node dissection, remained an option, the enlarged surgical scope, likelihood of postoperative complications, and probable requirement for adjuvant chemoradiotherapy in the event of margin positivity or nodal disease warranted consideration of alternative strategies. After fully informed and comprehensive counselling, the patient and her relatives elected to proceed with radiotherapy.

### 2.4 Radiotherapy Course

Radiotherapy began on October 14, 2024. Clinicians delineated treatment fields including the pelvic and superficial inguinal nodal regions specifically covering the bilateral common iliac bifurcation, internal and external iliac, obturator, deep inguinal, superficial inguinal, and presacral lymph nodes as well as the uterus, parametria, and entire vagina. The primary planning target volume (PTV) received 45 Gy in 25 fractions, and the nodal planning target volume (PTVnd) received a concomitant boost of 61.6 Gy in 28 fractions. Subsequently, intravaginal brachytherapy was initiated on November 22, 2024, administering 5 Gy per fraction over 5 fractions to the high-risk clinical target volume (HR-CTV) D90, and 4 Gy per fraction over 5 fractions to the intermediate-risk clinical target volume (IR-CTV) D90. Radiotherapy was completed on December 3, 2024. During the treatment course, the patient developed grade III leukopenia, which was effectively managed with supportive hematopoietic therapy.

### 2.5 Follow-up Imaging Findings

After 20 fractions of radiotherapy, pelvic MRI (11 November 2024; Fig. **4B**) showed a reduction of the dominant vaginal nodule from 20 mm at baseline to 16 mm, representing a 20% decrease in the longest diameter. HF-TRUS and CEUS (Fig. **2B**, **3B**) corroborated this finding, with Lesion 1 decreasing from 19 × 14 mm to 17 × 11 mm (15.2% reduction) and Lesion 2 decreasing from 19 × 14 mm to 16 × 11 mm (18.2% reduction) in Table **2**. Quantitative CEUS analysis revealed decreased perfusion, with area under the curve (AUC) values lower than pre-treatment measurements (Table **3**). The findings were judged compatible with early stable disease (SD) according to RECIST 1.1 criteria [6], and the patient continued treatment.

Six months after completion of radiotherapy, repeat MRI (16 June 2025; Fig. **4C**) revealed a residual 6 × 5 mm nodule in the right lower vagina (75% reduction from baseline). HF-TRUS (Fig. **2C**) demonstrated near-complete resolution of the previously noted anterior vaginal wall nodules, with Lesion 1 measuring 8 × 8 mm (51.5% reduction) and Lesion 2 completely resolved (100% reduction), with details in Table **2**. CEUS (Fig. **3C**) confirmed markedly decreased vascularity within the residual lesion. The concordant MRI and ultrasound data indicate that high-frequency transrectal linear-array ultrasound, combined with CEUS, supports the utility of ultrasound-based monitoring for radiotherapy response assessment.

The timeline of diagnosis and treatment is provided in Table **4**.

At the 6-month follow-up visit (16 June 2025), the patient reported no vaginal bleeding, discharge, dyspareunia, or pelvic pain. She denied any urinary or gastrointestinal symptoms. Physical examination revealed well-healed vaginal mucosa with no visible lesions or contact bleeding.

Written informed consent was waived by the Institutional Review Board of Women’s Hospital, School of Medicine, Zhejiang University (Ethics No: IRB-20240268-R), as this retrospective analysis used fully anonymized imaging data without identifiable patient features.

## 3 DISCUSSION

Vaginal malignant tumors are relatively uncommon in the literature; however, clinical experience suggests an increasing incidence with advanced age and menopausal status [7]. Our patient experienced abrupt post-menopausal vaginal bleeding 17 years after her final menstrual period, and pathological examination revealed high-grade squamous intraepithelial lesion (HSIL/VAIN III), signaling malignant transformation. Both pathology and imaging confirmed malignancy, an observation consistent with the reported rise in incidence of vaginal cancer after menopause [8]. Due to its rarity, evidence guiding diagnostic and surveillance strategies remains limited, and most management principles are extrapolated from cervical cancer literature [9]. This case report contributes to the limited body of evidence by demonstrating the potential utility of HF-TRUS with CEUS for monitoring radiotherapy response in vaginal cancer.

Early-stage vaginal lesions are easily overlooked on imaging, and the result is a diagnostic interval or delay. As the acknowledged gold standard for comprehensive evaluation, and with unrivalled soft-tissue contrast and multiplanar imaging capacity, MRI provides detailed soft-tissue characterization and anatomic delineation. It is thus preferred for mapping normal vaginal anatomy and pathological conditions and defining tumor extent, which ensures accurate diagnosis and treatment planning [10–13]. PET/CT capitalizes on the typically high metabolic activity of systemically staged vaginal cancer to reveal small lymph node metastases and distant lesions [14]. Imaging strategies should therefore be individualized based on patients’ clinical symptoms and diagnostic needs [15].

However, data on ultrasound-based alternatives remain sparse. Fischerova *et al*. [16] systematically reviewed the role of ultrasound in monitoring treatment response in gynecologic oncology, highlighting its potential for serial assessments during radiotherapy. Huang *et al*. [17] further showed that HF-TRUS with a linear array probe can resolve the layered architecture of the vaginal wall and permit accurate thickness measurements in both premenopausal and postmenopausal women. Zheng *et al*. [18] reported that when applied perpendicularly *via* the transrectal approach, the high-resolution linear-array probe enables accurate characterization of vaginal masses, including their size, extent, margins, morphology, and vascularity, with results comparable to magnetic resonance imaging. CEUS, using microbubble contrast agents, allows dynamic assessment of tumor microvascular perfusion and hemodynamic parameters [19]. CEUS yields tumor size measurements that correlate closely with MRI, and good agreement has been reported for cervical cancer invasion [20]. Our findings extend these observations to primary vaginal cancer, demonstrating that serial HF-TRUS measurements during radiotherapy correlated closely with MRI findings at corresponding time points, supporting the potential of this technique for treatment monitoring.

Follow-up evaluation is of paramount importance in the management of vaginal malignancies. It enables clinicians to detect progression and tailor treatment strategies in a timely manner. The prognosis of vaginal cancer is closely associated with early diagnosis and intervention, alongside factors such as tumor stage, histologic grade, and treatment response [21]. Several clinically relevant lessons emerge from this case. First, for patients in whom MRI is contraindicated (*e.g*., claustrophobia, metallic implants, renal impairment) or inaccessible due to cost or limited availability, HF-TRUS with CEUS offers a practical, office-based alternative for both initial tumor assessment and longitudinal surveillance. Second, the addition of CEUS provides functional information about tumor vascularity that complements morphological assessment. In this case, the “fast-in and equal-out” enhancement pattern and quantitative parameters (peak intensity, AUC, *etc*.) provided objective metrics for evaluating treatment response, with decreasing perfusion observed after radiotherapy. Third, anatomically, the vaginal cavity may harbor lesions that are easily overlooked, which often leads to late diagnosis. Studies suggest that vaginal distention with dry cotton balls or gel can enhance visualization. This simple technique, previously reported by Gardner *et al*. [13] for MRI using dry cotton balls or gel, can be readily adopted in clinical practice to enhance lesion delineation and reduce diagnostic uncertainty. The use of sterile ultrasound gel as an acoustic window in this case confirmed enhancement of subtle vaginal lesions detected by HF-TRUS.

In the present case, HF-TRUS was employed to assess therapeutic outcomes of the vaginal lesion. Results demonstrated that the efficacy was comparable to that of MRI. This supports the potential of HF-TRUS as a viable, cost-effective alternative for monitoring vaginal pathology and early diagnosis of vaginal lesions. Nevertheless, HF-TRUS has several limitations: limited penetration depth (3–5 cm), which restricts assessment of deeply invasive tumors (≥T2) and parametrial invasion; operator dependence, which leads to inter-observer variability; inability to assess pelvic lymph nodes compared to MRI/CT; and technical limitations, including gel artifacts and patient discomfort during examination. Meanwhile, as the results derive from a single-case report without controls or long-term follow-up, our findings are limited in generalizability and do not establish superiority of transrectal ultrasound for monitoring vaginal cancer response to radiotherapy. Immunohistochemistry results were not available for this case.

This case highlights several avenues for future investigation. Prospective cohort studies comparing HF-TRUS with CEUS to MRI in a larger series of vaginal cancer patients are needed to establish diagnostic accuracy metrics (sensitivity, specificity, positive and negative predictive values) and inter-observer variability. In addition, technical refinements such as three-dimensional HF-TRUS or fusion imaging with MRI may further enhance the utility of this technique for treatment planning and monitoring.

## CONCLUSION

By turning a thin layer of ultrasound gel into an acoustic window, HF-TRUS with CEUS resolves the individual anatomy of the vaginal wall and allows pre- and post-treatment assessment of characteristics such as tumor size, morphology, margins, and vascularity, as well as their spatial relationship to surrounding tissues. HF-TRUS with CEUS demonstrates superior utility in detecting vaginal pathology and monitoring responses to radiotherapy and chemotherapy. While delivering comparable diagnostic performance to magnetic resonance imaging, it offers advantages in ease of use, cost efficiency, and clarity in lesion delineation. Therefore, HF-TRUS with CEUS is a highly practical imaging tool that can facilitate early detection and treatment management of vaginal conditions.

## Figures and Tables

**Table 1:** Quantitative Contrast-Enhanced Ultrasound (CEUS) parameters of the lesions and reference tissues.

**Region**	**Arrival Time (s)**	**Peak Intensity (dB)**	AUC (dB*s)
**Lesion 1**	17.46	42.73	2983.04
**Lesion 2**	17.67	40.18	3037.52
**Thickened vaginal wall**	18.95	18.58	1237.39
**Reference area**	19.58	13.56	633.09

**Table 2:** Quantitative comparison of lesion dimensions and treatment response: HF-TRUS with CEUS *vs*. MRI

**Time Point**	**Modality**	**Lesion 1** **(mm)**	**Lesion 2** **(mm)**	**% Change**	**RECIST 1.1 Classification**
**Pre-treatment (Oct 7, 2024)**	HF-TRUS+CEUS	19 x 14	19 x 14	-	Baseline
**Pre-treatment (Oct 7, 2024)**	MRI	20 x 20	17 x 12	-	Baseline
**Mid-treatment (Nov 11, 2024)**	HF-TRUS+CEUS	17 x 11	16 x 11	Lesion1: -15.2% Lesion2: -18.2%	Lesion1:SD Lesion2:SD
**Mid-treatment (Nov 11, 2024)**	MRI	16 x 16	14 x12	Lesion1: -20% Lesion2: -10.3%	Lesion1:SD Lesion2:SD
**Post-treatment (Jun 16, 2025)**	HF-TRUS+CEUS	8 x 8	N/A	Lesion1: -51.5% Lesion2: -100%	Lesion1:PR Lesion2:CR
**Post-treatment (Jun 16, 2025)**	MRI	6 x 5	N/A	Lesion1: -72.5% Lesion2: -100%	Lesion1:PR Lesion2:CR

**Abbreviations:** SD: Stable Disease, PR: Partial Response, CR: Complete Response.

**Table 3:** Quantitative Contrast-Enhanced Ultrasound (CEUS) parameters of the lesions and reference tissues after 20 fractions of radiotherapy.

**Region**	**Arrival Time (s)**	**Peak Intensity (dB)**	AUC (dB*s)
**Lesion 1**	15.3	42.25	2824.03
**Lesion 2**	16.70	36.39	2182.30
**Thickened vaginal wall**	16.58	23.01	1644.53
**Reference area**	14.3	28.4	1985.57

**Table 4:** Timeline of diagnosis and treatment.

**Date**	**Clinical Event**	**Imaging / Treatment**	**Key Findings**
Sep 24, 2024	Initial presentation of post-menopausal bleeding for 20 days.	TVS (Transvaginal Ultrasound)	Identified an atrophic uterus and a 1.9 × 1.4cm vascular vaginal lesion.
Oct 7, 2024	Comprehensive pre-treatment staging and mapping.	HF-TRUS, CEUS & Pelvic MRI	Confirmed two hypervascular nodules (1.9 × 1.4 cm and 1.9 × 1.4 cm).
Sep 30, 2024	Pathological diagnosis *via* punch biopsy.	Histopathology	Confirmed HSIL (VAIN III) in the fornix and poorly differentiated SCC in the anterior wall.
Oct 14, 2024	Initiation of curative radiotherapy.	Treatment Planning (MDT)	Clinical Stage I (cT1N0M0); external-beam radiotherapy (EBRT) commenced.
Nov 11, 2024	Early response assessment (after 20 fractions of EBRT).	HF-TRUS, CEUS & Pelvic MRI	Demonstrated SD(Stable Disease) with a modest decrease in lesion size and AUC.
Nov 22, 2024	Transition to specialized radiotherapy phase.	Brachytherapy	Intravaginal brachytherapy initiated (5 Gy/fraction to HR-CTV).
Dec 03, 2024	Completion of the radiotherapy course.	Clinical Monitoring	Managed Grade III leukopenia with supportive hematopoietic therapy.
Jun 16, 2025	Six-month follow-up for long-term efficacy.	HF-TRUS, CEUS & Pelvic MRI	MRI showed only a residual 0.6 cm area; HF-TRUS showed a 0.8cm nodule.

## Data Availability

The data and supporting information are available in the article.

## LIST OF ABBREVIATIONS

HF-TRUS: High-frequency transrectal ultrasound
CDFI: Color Doppler Flow Imaging
CEUS: Contrast-enhanced ultrasound
HSIL: High-grade squamous intraepithelial lesion
VAIN: Vaginal intraepithelial neoplasia
MRI: Magnetic resonance imaging
PET/CT: Positron emission tomography/computed tomography
MDT: Multidisciplinary team
PTV: Planning target volume
PTVnd: Nodal planning target volume
HR-CTV: High-risk clinical target volume
IR-CTV: Intermediate-risk clinical target volume
DWI: Diffusion-weighted imaging
T2WI: T2-weighted imaging
RI: Resistance index
AUC: Area under the curve
HPV: Human papillomavirus
ASC-US: Atypical squamous cells of undetermined significance

## STANDARDS OF REPORTING

CARE guidelines were followed.
